# Comparative analysis of serum trace element levels in women with invasive cervical cancer in Lagos, Nigeria

**DOI:** 10.11604/pamj.2018.31.194.14425

**Published:** 2018-11-20

**Authors:** Kehinde Sharafadeen Okunade, Olayemi Olubunmi Dawodu, Omolola Salako, Gbemisola Eniola Osanyin, Adeyemi Adebola Okunowo, Rose Ihuoma Anorlu

**Affiliations:** 1 Department of Obstetrics & Gynaecology, College of Medicine, University of Lagos, Nigeria; 2 Department of Anatomic and Molecular Pathology, College of Medicine, University of Lagos, Nigeria; 3 Department of Radiation Oncology, Lagos University Teaching Hospital, Nigeria

**Keywords:** Cervical cancer, Lagos, LUTH, trace elements

## Abstract

**Introduction:**

Trace elements although present in minute quantities in human blood, they play a vital role in many biochemical enzymatic reactions and have been examined critically as a potential key factor in various human diseases including cancers. This study was aimed to determine the association between serum levels of trace elements and invasive cancer of the cervix.

**Methods:**

This was an analytical cross-sectional study carried out among women seen at the Lagos University Teaching Hospital (LUTH). Fifty histologically diagnosed patients with squamous cells carcinoma of the cervix, who had not had any treatment and 100 cancer-free volunteers were recruited. A structured interviewer-administered questionnaire was used to collect relevant data following which venous blood sample was obtained from each participant. Serum zinc, copper and selenium concentrations were then measured. The associations of serum trace elements and invasive cervical cancer were tested using the independent sample t-test. All significances were reported at P<0.05.

**Results:**

There were significantly low serum levels of zinc and selenium in cervical cancer patients with no significant difference seen in the serum level of copper among cervical cancer patients compared to their cancer-free control counterparts.

**Conclusion:**

These alterations in trace elements levels may be important in the pathogenesis of cervical cancers; however, future robust prospective studies are needed to determine if routine provision of these supplements will result in improved cervical cancer treatment outcomes in Nigerian women.

## Introduction

Cervical cancer is the second most common cancer among women in the developing countries and the seventh most common cancer in the developed countries [1]. Over 500,000 new cases are seen yearly [1] with over 80% of them being from the developing countries [1, 2]. Worldwide, it claims the lives of 300,000 women annually with over 80% coming from the developing countries [1]. It is the most common gynaecological cancer and a leading cause of cancer deaths among women in Nigeria [3]. Out of the estimated 14,550 women who are diagnosed with the disease in Nigeria annually, 9,659 will die from it [4]. Accumulation of free radicals has been implicated in the pathogenesis of many diseases [5]. Trace elements although present in minute quantities in human blood, play a vital role in many biochemical enzymatic reactions and have been examined critically as a potential key factor in various human diseases including cancers. They are known to play a pivotal role in the process of normal growth and differentiation of various tissues in animals and humans [6]. Their requirement for sustenance of tumour cell proliferation is hence considered to be of significant importance [7]. Hence their roles in the prevention of cervical cancer and other invasive cancers of humans are now subjects of discussions and intensive research interests [8-10]. Zinc is one such essential element that prevents the formation of free radicals. In addition to its role as an anti-oxidant, zinc is also known to participate in nearly 120 reactions taking place in a living organism [6].

More recently, studies have shown that this element may also play an important regulatory role in initiation of cell-mediated immunity [11]. Recent studies have demonstrated that Zinc deficiency seriously inhibited the development of lymphoid organs, impaired the progression of lymphocytes from the G0/G1 phase to the S phase and caused pathological injury in the lymphoid organs [12]. Significant changes have also been observed for serum copper concentration in various malignant conditions [13] and as a result serum copper concentration is considered as a non-specific marker for monitoring the progression of malignant disease [14]. The essential trace element, Selenium, is one of the major non-enzymatic endogenous antioxidants in human body. The interest of oncologists is now in its roles as a radioprotection of normal tissues, radio-sensitizer in malignant tumours, anti-oedematous effect, prognostic impact and its effects in primary and secondary cancer prevention [15]. Selenium is a constituent of the small group of selenocysteine-containing selenoproteins and elicits important structural and enzymatic functions [16]. It has been shown to possess cancer-preventive and cytoprotective activities in both animal models and humans. It is well established that Selenium has a key role in redox regulation and antioxidant function and hence in membrane integrity, energy metabolism and protection against DNA damage [17]. The serum levels of zinc, copper and selenium in invasive cervical cancer patients have been investigated by a good number of researchers both within and outside Nigeria, with variations in the reported findings [6, 17-19]. There are however, still limited data available to show the association between these trace elements and invasive cancer of the cervix among Nigerian women. This hospital based study was therefore aimed to evaluate the levels of these important trace elements in Nigerian women with invasive cervical cancer.

## Methods

This was an analytical cross-sectional study carried out among women seen at the cytology and gynaecology out-patient clinics of the Lagos University Teaching Hospital (LUTH), Lagos, Nigeria. LUTH is an over 1000 bedded teaching hospital located in the Central Lagos metropolis in South-West Nigeria. The hospital provides services to patients from the neighbouring South-Western states. It is the largest in the state and offers mainly clinical services among which include gynaecological oncology services. The gynaecology clinic is an-all female clinic with the cytology clinic being an off-shoot of it. In addition to receiving patients from the gynaecology clinic, the cytology clinic is the meeting point for all women from all other clinics of the hospital referred for routine cytological evaluation. The sample size (*N*) for the study was calculated using the statistical formula by Schlesselman [20]. Participants for the study were grouped as follows: 50 recently diagnosed patients with squamous cells carcinoma of the cervix and 100 control subjects who had no malignancy. Eligible subjects for the case group were women with biopsy proved cases of squamous cells carcinoma of the cervix; that had not undergone any treatment i.e. surgery, chemotherapy or radiotherapy; who did not suffer from any major illness in the past; who had not taken long course of any mineral supplement during the last six months. The control subjects were those who were not suffering from any cancerous lesions. The case and control subjects belonged to the same socio-economic status and same diet habits. Informed written consent was obtained from each participant upon explanation of the nature and purpose of the study. A structured interviewer-administered questionnaire was then used to collect relevant data. A volume of 5ml venous blood samples were obtained by venepuncture and collected in Ethylenediaminetetraacetic acid (EDTA) bottle. Standard precautions for trace element determination were taken and samples with signs of haemolysis discarded. Serum Zinc and Copper concentrations were estimated using direct atomic absorption spectrophotometer [21]. Selenium concentration was measured using the hydride generation method [22]. Serum was digested by a mixture of nitric and perchloric acid. After hydride generation and using a sodium borohydride method, the selenium concentration is then determined (AAS Model ECI 4141). All data were entered in the computer and analysed using SPSS version 22.0 statistical package for windows manufactured by IBM Corp, Armonk, NY, United States. Descriptive statistics were then computed for all continuous data and expressed as mean and standard deviation. The associations between any two groups of continuous variables were tested using the independent sample t-test. Statistical significance was defined as P<0.05.

**Ethical approval:** Ethical approval for the study was obtained from the hospital's Health Research and Ethics committee prior to the commencement of the study and the ethical principles according to the Helsinki declaration were considered during the course of the research.

## Results

**Figure 1:**
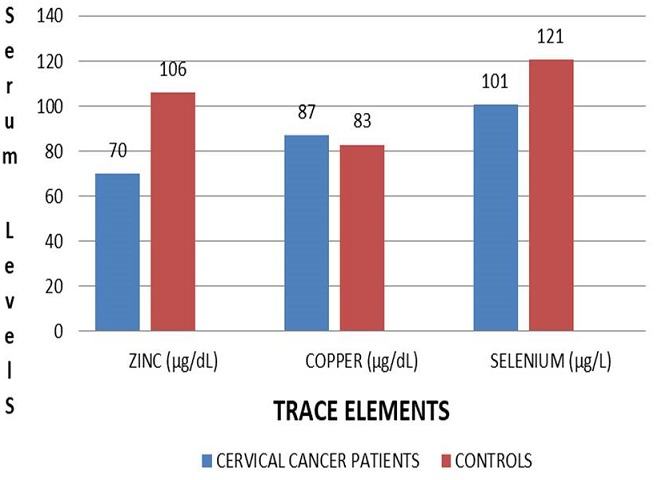
Comparisons of mean serum levels of the trace elements in the cervical cancer patients and their controls

The demographic characteristics of the study participants are presented in Table 1 and there were no statistically significant differences between patients with cervical cancer and their control counterparts with regards to age (P=0.177), BMI (P=0.093), and total albumin levels (P=0.134). However there was a significant difference in the haemoglobin levels between the two groups of participants (P=0.042) The results as presented in Figure 1 showed the comparisons between the mean serum levels of trace elements in participants having squamous cells carcinoma and their cancer-free controls.

**Table 1:** Demographic characteristics of study participants

Demographic characteristics	Patients (n=50)	Controls (n=150)	P-value
	Mean ± SD	Mean ± SD	
Age (years)	43.1 ± 7.5	40.9 ± 11.8	0.177
BMI (kg/m^2^)	23.6 ± 3.1	22.7 ± 6.3	0.093
Haemoglobin level (g/dL)	8.4 ± 2.6	12.7 ± 1.4	0.042
Total albumin (g/dL)	5.1 ± 1.3	4.9 ± 2.2	0.134

*BMI – Body Mass Index; SD – Standard Deviation

**Zinc:** The mean serum level of zinc was significantly lower in the cervical cancer group than the cancer-free control group (70.1 ± 11.7 μg/dL vs. 105.8 ± 16.5 μg/dL; P=0.003).

**Copper:** The mean level was higher in the cervical cancer patients but there was no statistically significant difference in the level in serum of patients as compared to its level in normal control group of individuals (86.6 ± 15.5 μg/dL vs. 82.8 ± 20.3 μg/dL; P=0.099).

**Selenium:** There was a significantly lower mean selenium levels in the cervical cancer patients compared to the control participants (101.3 ± 7.7 μg/L vs. 120.9 ± 18.3 μg/L; P=0.026).

## Discussion

In this study, we investigated the levels of three serum trace elements in patients with histologically diagnosed cervical cancer compared with cancer-free controls. Our study found a reduction in haemoglobin levels among participants with histologically diagnosed cervical cancer and this has been explained to occur as a result of iron deficiency and tumour bleeding in these patients [19, 23]. The findings from our present study indicated a strong association of low serum levels of Zinc and Selenium with invasive squamous cells carcinoma of the cervix. Previous studies, just like this current study, have also reported that serum Zinc concentrations were decreased in patients with ovarian, testicular, cervical, bladder and renal cancer [24-28]. Zinc plays an anti-carcinogenic role through structural stabilization of deoxyribonucleic acid (DNA), ribonucleic acid (RNA), and ribosome. It has a protective effect against free-radical injury [29]. Our study just like some other epidemiologic studies revealed that a low selenium level in serum increase the risk of human cancers such as cancer of the stomach, oesophagus, colon, lung, prostate and breast [30]. It has been suggested that selenium protects cell by inhibiting free oxygen radical production. Moreover, an important antioxidant Vitamin E is transported by selenoproteins. Selenium has been shown to possess cancer-preventive and cytoprotective activities in animal models and humans [15, 31]. In our study, even though the mean serum concentration of Copper in the cancer patients was higher than in the controls, we did not find any statistically significant association. Copper plays a role in the production of haemoglobin, myelin, collagen and melanin as an essential nutrient [32] and studies have shown that normal immune function requires adequate Cu intake [7, 31, 32]. However, serum copper values are significantly elevated in many disease conditions such as chronic obstructive pulmonary disease (COPD), malignancies and psychosis [33]. The major limitations to this study were that it was hospital-based and thus the findings may not be generalizable to the public and also the study design will not allow us to conclude if Zinc and/or Selenium deficiencies actually preceded or occurred as a result of cervical cancer.

## Conclusion

The study found significantly lower concentrations of zinc and selenium in cervical cancer patients. Zinc and selenium supplements may therefore result in reduced cervical cancer occurrence among high risk Nigerian women. However, future robust prospective studies are needed to determine if these trace element concentrations will impact clinical outcomes and to establish whether routine provision of these trace elements as supplements will result in improved cervical cancer treatment outcomes in Nigerian women.

### What is known about this topic

- That cervical cancer is the most common gynaecological cancer and a leading cause of cancer death in women in Nigeria;
- Trace elements although present in minute quantities in human blood, play a vital role in many biochemical enzymatic reactions and have been examined critically as a potential key factor in various human diseases including cancers;
- The roles of trace elements in the prevention of cervical cancer and other invasive cancers of humans are now subjects of discussions and intensive research interests.

### What this study adds

- The study found significantly lower concentrations of zinc and selenium in cervical cancer patients to their cancer-free control counterparts;
- There is, however, a relatively low level of practice of cervical cancer prevention among the respondents;
- The study, therefore, propose that routine provision of zinc and selenium supplements may result in reduced cervical cancer occurrence among Nigerian women.

## Competing interests

The authors declare that competing interests.
